# Pancreatic Stellate Cells and the Targeted Therapeutic Strategies in Chronic Pancreatitis

**DOI:** 10.3390/molecules28145586

**Published:** 2023-07-22

**Authors:** Man Chang, Wenjuan Chen, Ruting Xia, Yangyue Peng, Pandi Niu, Hui Fan

**Affiliations:** 1 Guangdong Metabolic Diseases Research Center of Integrated Chinese and Western Medicine, Guangzhou 510006, China; 2112048136@gdpu.edu.cn (M.C.);; 2 Guangdong TCM Key Laboratory for Metabolic Diseases, Guangzhou 510006, China; 3 Key Laboratory of Glucolipid Metabolic Disorder, Ministry of Education of China, Guangzhou 510006, China; 4 Key Unit of Modulating Liver to Treat Hyperlipemia SATCM (State Administration of Traditional Chinese Medicine), Guangzhou 510006, China; 5 Institute of Chinese Medicine, Guangdong Pharmaceutical University, Guangzhou 510006, China

**Keywords:** chronic pancreatitis, pancreatic fibrosis, pancreatic stellate cells, therapeutic strategies

## Abstract

Chronic pancreatitis (CP) is a disease characterized by inflammatory recurrence that accompanies the development of pancreatic fibrosis. As the mystery of CP pathogenesis is gradually revealed, accumulating evidence suggests that the activation of pancreatic stellate cells (PSCs) and the appearance of a myofibroblast-like phenotype are the key gatekeepers in the development of CP. Targeting PSCs to prevent their activation and conversion to a myofibroblast-like phenotype, as well as increasing antioxidant capacity to counteract ongoing oxidative stress, are effective strategies for preventing or treating CP. Therefore, we reviewed the crosstalk between CP and pancreatic fibrosis, summarized the activation mechanisms of PSCs, and investigated potential CP therapeutic strategies targeting PSCs, including, but not limited to, anti-fibrosis therapy, antioxidant therapy, and gene therapy. Meanwhile, the above therapeutic strategies are selected in order to update the available phytopharmaceuticals as novel complementary or alternative approaches for the prevention and treatment of CP to clarify their potential mechanisms of action and their relevant molecular targets, aiming to provide the most comprehensive therapeutic treatment direction for CP and to bring new hope to CP patients.

## 1. Introduction

Chronic pancreatitis (CP) is a disease characterized by recurrent episodes of pancreatic inflammation leading to the development of fibroinflammatory histology, with clinical manifestations of abdominal pain, steatorrhea, pancreatic exocrine and endocrine insufficiency, and imaging-detectable pancreatic injury [1]. The estimated incidence of this disease is 8–12 per 100,000 people worldwide, and the prevalence is up to 36.9–52.4 per 100,000 individuals. The median survival time for CP patients is 15–20 years [2]. In addition, CP is considered a risk factor for pancreatic ductal adenocarcinoma (PDAC), with a relative risk 13.3% higher than that of the general population [3]. Therefore, CP can be regarded as a precancerous lesion associated with PDAC to some extent.

However, the pathogenesis of CP is not yet well defined, and effective treatment strategies are lacking [1]. Currently, it is believed that recurrent oxidative stress or inflammation are the underlying causes of persistent pancreatic injury, leading to irreversible changes in pancreatic structure and function and ultimately, to the occurrence of CP [4]. Therefore, we reviewed CP and its pathogenesis and summarized the prevention or treatment strategies that have recently received increasing attention, aiming to provide a comprehensive perspective to unveil the mysterious mechanism of CP and bring hope to CP patients.

## 2. Chronic Pancreatitis and Pancreatic Fibrosis

### 2.1. Risk Factors and Pathogenesis of Chronic Pancreatitis

**Figure 1:**
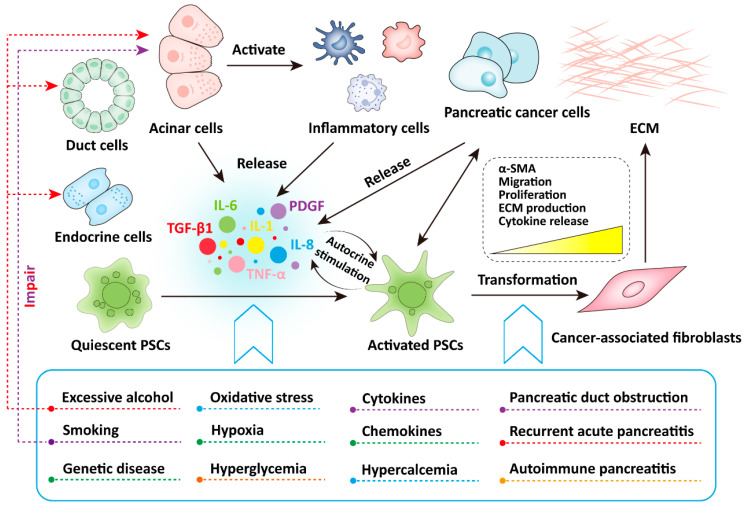
Risk factors for chronic pancreatitis and the activation factors of pancreatic stellate cells in chronic pancreatitis.

Excessive alcohol consumption is the most common cause of CP, with approximately 70% of CP cases caused by alcohol abuse [1,5]. For example, the risk of CP is significantly higher in chronic drinkers than in non-drinkers [6]. In addition, smoking is also a high-risk factor for CP, and the risk of CP caused by smoking exhibits a dose-dependent effect [1,7]. Quitting smoking or alcohol consumption, or both, significantly reduces the risk of CP progression [8]. In addition, other etiological risk factors include genetic diseases, pancreatic duct obstruction, recurrent acute pancreatitis, autoimmune pancreatitis, hypertriglyceridemia, hypercalcemia, IgG4-related diseases, chronic kidney disease, and unknown mechanisms [5,9] (Figure 1). In summary, according to current limited understanding, the occurrence and development of CP are mediated by multiple risk factors [10].

There are three main cell types in the pancreas: endocrine cells that produce various hormones in the islets, acinar cells that produce digestive enzymes, and pancreatic duct cells that form ducts [11]. In vitro experiments highlight the accumulation of reactive oxygen species (ROS) as a trigger and enhancer of inflammation, as ROS activates signaling cascades and converts damaged acinar cells into production sites for chemokines and cytokines [12]. Increased ROS were observed in CP, which increases the risk of pancreatic cancer through the activation of pancreatic stellate cells (PSCs) [13]. Ethanol damages acinar cells, pancreatic ductal cells, and PSCs through the oxidative metabolite acetaldehyde and the non-oxidative metabolite fatty acid ethyl ester [13]. The nicotine in tobacco produces toxic metabolites in the body and can also cause damage to the acinar cells [13] (Figure 1).

### 2.2. Chronic Pancreatitis and Pancreatic Fibrosis

The pathology of CP is characterized by inflammatory cell infiltration, alveolar atrophy, and pancreatic fibrosis. Pancreatic fibrosis is a histological change accompanied by chronic or recurrent acute episodes of pancreatic injury and inflammation. It is a pathological process in which cellular injury and inflammatory cell infiltration are the initial events, multiple cytokines and inflammatory mediators are involved, and complex signaling pathways mediate the activation of PSCs and the production of the extracellular matrix (ECM). Due to the increase in lipid peroxidation products and the release of mast cell degranulation products, fibrosis is a marker indicating that interstitial PSCs have been activated in CP [14]. Pancreatic fibrosis is considered to be a pathological hallmark of CP and PDAC, especially in cases of advanced disease where replacement of normal pancreatic parenchyma by fibrosis is a key component of endocrine and exocrine pancreatic insufficiency [14]. Therefore, it is evident that PSCs is associated with CP, and that targeting PSCs may be a highly effective prospective therapeutic strategy for CP.

## 3. Activation of Pancreatic Stellate Cells Is the Gatekeeper of Chronic Pancreatitis

### 3.1. Pancreatic Stellate Cells and Their Activation Mechanisms

Early studies regarding different cell populations in the pancreas have shown that the pancreatic resident and quiescent fibroblast populations are scattered between the pancreatic lobules and the surrounding regions of acini, storing retinol-rich cytoplasmic lipid droplets and expressing desmin, similar to the behavior of hepatic stellate cells (HSCs); hence, they are called pancreatic stellate cells (PSCs) [15,16]. PSCs are pluripotent cells, accounting for approximately 4–7% of parenchymal cells, and playing a key role in maintaining the structure of connective tissue [17,18]. Under normal physiological conditions, PSCs maintain their quiescent state by expressing nestin, vimentin, glial fibrillary acidic proteins, and desmin. The quiescent PSCs exhibit many typical characteristics, such as abundant perinuclear lipid droplets, molecular markers (cytoglobin and lipophilins), and a low capacity for proliferation, migration, and synthesis of ECM [19,20]. In addition, the quiescent PSCs play a role in the storage of vitamin A, immunity, and the protection of normal pancreatic structures [21].

It is currently believed that multiple factors can lead to the activation of quiescent PSCs, such as smoking, alcohol consumption, oxidative stress, hypoxia, hyperglycemia, cytokines, chemokines, and in vitro culture [22]. The activated PSCs exhibit a myofibroblast-like phenotype: PSCs lose cytoplasmic lipid droplets and begin to express α-smooth muscle actin (α-SMA), cytokines, and ECM (including collagen I, collagen III, hyaluronic acid, and fibronectin), while cell migration and proliferation are enhanced [15,16,23] (Figure 1). In addition, the mRNA expression level of *MMP-3* in activated PSCs is significantly upregulated, and the mRNA expression levels of basement membrane component IV-α collagen protein is significantly downregulated, which may contribute to recombine the activated form of ECM [24]. Notably, the activation of PSCs can also be induced by pathological conditions. For example, the quiescent PSCs are continuously activated in diseases such as CP or PDAC [23,25]. Thus, activated PSCs is recognized to be the responsible for excessive pancreatic fibrosis.

The risk factors for CP include excessive alcohol consumption, smoking, genetic diseases, pancreatic duct obstruction, recurrent acute pancreatitis, autoimmune pancreatitis, hypertriglyceridemia, hypercalcemia, and unknown mechanisms. Among these, excessive alcohol consumption leads to impairment of the acinar cells, pancreatic duct cells, and endocrine cells, while smoking also leads to damage to the acinar cells. In addition, multiple factors, including smoking, alcohol consumption, oxidative stress, hypoxia, hyperglycemia, cytokines, and chemokines, often lead to the activation of quiescent PSCs. After the pancreas is damaged by the aforementioned factors, the damaged acinar cells can activate inflammatory cells to release pro-inflammatory cytokines, which activate quiescent PSCs through paracrine stimulation. The activated PSCs exhibit a myofibroblast-like phenotype, causing the expression of α-SMA, cell proliferation, cell migration, and ECM production. The activated PSCs can also release cytokines and continuously activate them by autocrine stimulation, leading to pancreatic fibrosis.

### 3.2. Activation of Pancreatic Stellate Cells Mediates the Occurrence and Development of Chronic Pancreatitis

As previously described, activated PSCs secrete excessive amounts of ECM, leading to inter- and intralobular fibrosis, and continued crosstalk between PSCs and ECM further enhances the stiffness of ECM. Therefore, the activation of PSCs is considered central to pathological pancreatic fibrosis in CP [1,25]. The progression of CP is clinically observable, starting with damage to the acinar cells, followed by β-cell dysfunction, and finally, by a decrease in α-cell function, which marks the end stage of the disease. In the early stages of CP, damaged acinar cells activate key inflammatory cells including macrophages, granulocytes, and lymphocytes. Subsequently, all these cells release large amounts of pro-inflammatory cytokines such as IL-1, IL-6, IL-8, TNF-α, TGF-β1, and platelet-derived growth factor (PDGF), which can activate the PSCs through paracrine stimulation [13] (Figure 1). Moreover, activated PSCs can also secrete cytokines that continuously activate PSCs through autocrine stimulation, and the continuous activation of PSCs leads to a higher synthesis rate of the extracellular matrix (ECM) than its degradation rate, ultimately leading to pancreatic fibrosis [26,27,28] (Figure 1). Among cytokines, TGF-β1 is the most important driver of pancreatic fibrosis, promoting the activation of PSCs and the production of ECM [28,29,30] (Figure 1). In addition, NF-κB and AP-1 play an important role in initiating the inflammatory cascade and the necroinflammatory response in CP [27,31].

Recently, macrophages in the progression of CP have received widespread attention. Macrophages are differentiated monocytes that can be activated and polarized into different types by classical or bypass ligands, thereby mediating further targeting effects, and these have been shown to play a key role in immunity, inflammation, and oncology [32,33,34]. Under normal physiological conditions, the number of resident macrophages in the pancreas is relatively low, and only when pathological changes occur will a large number of macrophages engage in phagocytosis, polarization, and repair. Previous in vivo studies have shown that macrophages and T cells are the main immune cell types in the pancreas of CP patients [27,28,35,36]. Damage to pancreatic acinar cells leads to macrophage-induced infiltration of inflammatory cells, and inflammatory cells can activate PSCs and induce pancreatic fibrosis. In addition, macrophages can also affect the crosstalk between islet cells and PSCs [37,38,39]. For example, the pancreas in the mouse CP model is infiltrated by M2 macrophages instead of M1 macrophages, and M2 macrophages can effectively activate PSCs through a “feed-forward” mechanism, suggesting that macrophages play a crucial role in the process of pancreatic fibrosis [40]. In addition, an increase in lymphocytes was observed in pancreatic tissue samples from CP patients, where CD8^+^ T cells located between the pancreatic parenchyma and the fibrotic region were recognized to be a key factor in disease severity, and the cytotoxicity mediated by CD8^+^ T cells or NKT cells may play a critical role in the pathogenesis of CP [41]. It is worth noting that mast cells, dendritic cells, eosinophils, monocytes, and B cells are also involved in the inflammatory response in CP [27].

**Figure 2:**
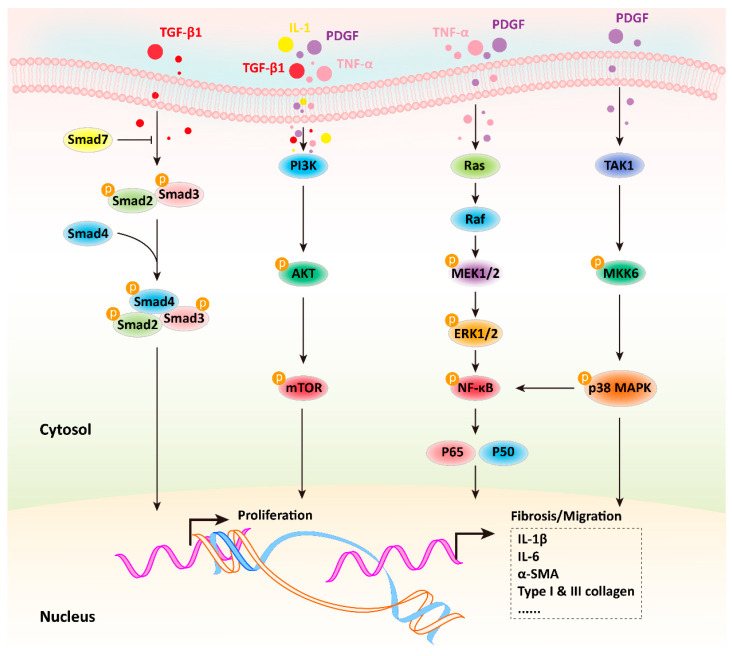
The potential mechanism of PSCs activation to form a myofibroblast-like phenotype and lead to pancreatic fibrosis in chronic pancreatitis.

Accumulating evidence suggests that the activation of PSCs and the development of CP involve several important signaling pathways, including Smad, PI3K-AKT, MAPK, and p38-MAPK [26] (Figure 2). Further information is available from the highly commendable review authored by Guihua Jin et al. [26]. In addition, it has been shown that the JAK/STAT pathway is essential for the proliferation and activation of PSCs, and inhibiting this pathway can reduce caerulein-induced CP in vivo [42]. TGF-β1 induces the activation of the NF-κB pathway by regulating the expression of p-TAK1 in PSCs [43]. A recent study showed that enhanced lipoprotein metabolism caused by the very low density lipoprotein receptor in PSCs increases the degree of fibrosis, in which IL-33 is a key factor mediating CP [44]. In addition, oxidative stress is also considered to be one of the most important mechanisms in the pathogenesis of CP. Repetitive oxidative stress in the pancreas converts fat-storing PSCs into myofibroblast-like cells that can produce ECM, chemokines, and adhesion molecules in response to inflammatory infiltration [45]. Epithelial mesenchymal transition (EMT) is required for many physiological developmental steps; however, EMT contributes to tumorigenesis and metastasis [46]. During tumorigenesis, PSCs transforms into an active myofibroblast-like phenotype and are involved in multiple processes. PSCs secrete metalloproteinases (MMPs) and matrix metalloproteinases (TIMPs), including MMP2, MMP9, MMP13, TIMP1, and TIMP2, indicating that PSCs help maintain the balance of the ECM in a healthy pancreas [12]. However, the activation of PSCs in CP and pancreatic cancer can disrupt this balance [21]. The epidermal growth factor receptor (EGFR) pathway is involved in pancreatic fibrosis, and the overexpression of heparin-binding epidermal growth factor-like HB-EGF in pancreatic islets is considered to be one of the mechanisms leading to extensive pancreatic fibrosis during the CP process [21]. PSCs express HB-EGF-activated EGFR and promote PSCs activation and migration in an autocrine manner [21]. All of these indications suggest that the activation of PSCs is a key gatekeeper for the occurrence and development of CP, and targeting PSCs has become the most promising strategy for the prevention and treatment of CP.

After the pancreas if impaired, the pro-inflammatory cytokines are released from the acinar cells, inflammatory cells, and activated PSCs, leading to a series of signaling cascades that result in activated PSCs exhibiting a myofibroblast-like phenotype, ultimately leading to pancreatic fibrosis. Among these, the Smad signaling pathway is mainly activated by TGF-β1; the PI3K-AKT signaling pathway can be activated by TNF-α, TGF-β1, IL-1 and PDGF; the MAPK signaling cascade is activated by TNF-α and PDGF; and the p38 MAPK signaling pathway can be activated by PDGF. The activation of all these signaling pathways leads to the activation of PSCs and the development of pancreatic fibrosis in CP.

## 4. Therapeutic Strategies of Chronic Pancreatitis Targeting Pancreatic Stellate Cells

As previously mentioned, the activation of PSCs is the gatekeeper of CP. Targeting PSCs to prevent or treat CP has emerged as a widely accepted novel therapeutic strategy. However, the mechanism by which PSCs are activated is unclear, leading to a lack of effective intervention measures to prevent or combat inflammation and fibrosis in CP. Thus, this section focuses on different therapies targeting PSCs for the treatment of CP, including, but not limited to, anti-fibrosis therapy, antioxidant therapy, and gene therapy (Table 1).

### 4.1. Anti-Fibrosis Therapy

Until now, plant ingredients and natural chemicals have been widely used as complementary and alternative medicines to extend lifespan and treat diseases [51]. The natural compounds in plants also show promising applications in the prevention and treatment of CP. Based on the anti-fibrosis therapies targeting PSCs, we summarized the phytochemicals that showed potential anti-fibrosis effects on PSCs, including, but not limited to, curcumin, resveratrol, rhein, emodin, epigallocatechin gallate, ellagic acid, embelin, eruberin A, and metformin.

#### 4.1.1. Curcumin

Curcumin is a turmeric polyphenol derived from the turmeric rhizome, with antioxidant and anti-inflammatory properties. In vitro and in vivo studies have demonstrated the anti-fibrotic activity of curcumin in CP and pancreatic cancer models. For example, curcumin inhibited the PDGF-induced proliferation of PSCs and reduced the expression of α-SMA, IL-1 β, and TNF-α induced MCP-1 production, type I collagen production, and AP-1 activation in activated PSCs [47]. Curcumin inactivates PSCs by inhibiting their proliferation, and mechanistically, the inhibition of ERK1/2 activation and the upregulation of heme oxygenase-1 (HO-1) synergistically increase cellular carbon monoxide levels, thereby activating p38 MAPK, leading to a decreased proliferation of PSCs [48]. In addition, curcumin and three phenolic compounds can inhibit the mRNA and protein levels of the fibrosis mediators α-SMA, type I collagen, and fibronectin in TGF-β-activated primary PSCs, and their potential mechanisms were associated with the downregulation of the NF-κB signaling pathway [12]. In addition, a newly synthesized curcumin analogue, L49H37, more effectively induced apoptosis in PSCs at a concentration 10-fold lower than that of curcumin [78]. Notably, curcumin had a direct effect on pancreatic β-cells; it reduced the volume of pancreatic β-cell in activated PSCs [79]. This evidence suggests that curcumin may be used as an anti-fibrotic agent to treat pancreatic fibrosis and PSCs-related pathologies, including PDAC [51].

#### 4.1.2. Resveratrol

Resveratrol is a polyphenol stilbene found in high quantities in grapes, raspberries, blueberries, cocoa, and peanuts [80,81]. Several in vitro studies have demonstrated the excellent effects of resveratrol in the treatment of CP. For example, resveratrol induces apoptosis and attenuates fibrosis in CP by promoting caspase-3 activation [50]. It also inhibits the markers of PSCs activation, such as α-SMA, collagen I, and fibronectin, by downregulating NF-κB signaling [51].

#### 4.1.3. Rhein

Rhein is a natural anthraquinone derivative with antibacterial, anti-inflammatory, anti-angiogenic, and anticancer properties [82,83,84,85,86,87,88,89]. Among these, the anti-fibrotic activity of rhein has been demonstrated in the CP model. In a cerulein-induced CP mouse model, daily treatment with 50 mg/kg rhein significantly attenuated fibrogenesis by reducing the immunoreactivity of fibrosis activators, including α-SMA and TGF-β, in pancreatic tissue and reduced fibronectin and type 1 collagen deposition in exocytosis [53]. In addition, rhein attenuated PSCs activation and inhibited sonic hedgehog SHH/GLI1 signaling, while also inhibiting α-SMA, fibronectin, and MMP in PSCs by modulating the SHH pathway [53,54]. Mechanically, the downregulation of NF-κB and STAT3 signaling pathways may be a potential mechanism for the anti-fibrotic and anti-tumor effects of rhein [51,90]. In addition, 20 μM rhein can significantly reduce the NF-κB subunit involved in inflammatory responses in PSCs [51].

#### 4.1.4. Emodin

Emodin exhibits anti-inflammatory, anti-angiogenic, anti-dyslipidemic, and anti-cancer properties [91,92]. The treatment of PSCs with 4 μM emodin can significantly downregulate the expression of fibrosis markers α-SMA, fibronectin, and collagen I, thereby reducing the cell viability of primary PSCs [51]. In addition, emodin exhibits strong anti-cancer activity in pancreatic cancer through various mechanisms such as targeting cell proliferation and inducing cell apoptosis [51,93].

#### 4.1.5. Epigallocatechin Gallate

Polyphenols in green tea can prevent fibrosis by reducing the activation of PSCs [56]. Epigallocatechin gallate (EGCG) is the main phenolic compound in green tea, exhibiting antioxidant, anti-inflammatory, and anti-cancer properties [94,95], and it has been shown to inhibit PDGF-induced PSCs proliferation and migration [55]. Ethanol increases the protein expression level of α-SMA, activates TGF-β1, and induces p38 MAPK phosphorylation. EGCG treatment completely eliminates the phosphorylation of p38 MAPK, thereby inhibiting the ethanol-induced activation of PSCs and the transformation to a myofibroblast-like phenotype [56,57].

#### 4.1.6. Ellagic Acid

Ellagic acid is a non-flavonoid polyphenol of plant origin, mainly found in berries, grapes, pomegranates, and walnuts [12]. It has been shown that ellagic acid has a significant anti-fibrotic activity in the CP model. Ellagic acid treatment significantly eliminated the development of pancreatic fibrosis and reduced the mRNA expression levels of α-SMA and TGF-β1 in a CP rat model. At the same time, ellagic acid inactivated PSCs by reducing protein levels of α-SMA and ECM type I and III procollagen, preventing the transformation of PSCs from a quiescent state to a myofibroblast-like phenotype [58]. In addition, ellagic acid inhibited PDGF-BB-induced PSCs proliferation and migration [59]. Mechanically, ellagic acid reduces the activation of the downstream signaling pathways of c-Raf/MAPK/ERK and PI3K/AKT, which are important for PSCs cell proliferation and migration [58]. 

#### 4.1.7. Eruberin A

Eruberin A, an organic flavanol glycoside, has been shown to exhibit anti-fibrotic activity against primary PSCs. Treatment of LTC-14 cells with 20 μg/mL eruberin A resulted in a dose-dependent decrease in the growth rate and a significant inhibition of the gene expression of major fibrotic filaments and ECM mediators, including smooth muscle α-actin, collagen type I-α 1, and fibronectin 1 [60]. Furthermore, eruberin A can inhibit NF-κB activation and SHH signaling, as well as the activation of the PI3K/AKT pathways associated with the downstream cascades of inflammation and fibrogenesis [60].

#### 4.1.8. Vitamin A/D and Their Derivatives

Vitamin A (VA) derivatives induce significant apoptosis, inhibit proliferation, and suppress PSCs production of ECM in vitro. Moreover, the vitamin A analogs can inhibit PSCs activation in pancreatic ductal adenocarcinoma [61]. Another VA analogue, ATRA, can reactivate the quiescent state of PSCs in KPC mice [96]. These studies suggest a potential role for VA analogs in the treatment of CP-related fibrosis.

The high prevalence of vitamin D (VD) deficiency in CP patients is associated with the risk and prognosis of CP [97,98]. VD and its derivatives have now been shown to alleviate pancreatic fibrosis by inhibiting PSCs activation and reducing ECM deposition. VD analogs may restart the quiescent state of the PSCs and reduce fibrosis in CP [62,63]. In addition, VD can increase lipid droplet storage, inhibit PSCs activation, and reduce the expression of α-SMA and IL-6 [63]. Exposure of activated PSCs to different physiological concentrations of 1,25(OH)_2_D_3_ revealed that 1,25(OH)_2_D_3_ inhibited the expression of fibronectin and collagen type I-α 1, along with the proliferation of PSCs, and the anti-proliferative capacity was positively correlated with 1,25(OH)_2_D_3_ concentration [35]. This evidence suggests that VD may be an effective antifibrotic therapy for targeting PSCs in CP. Additionally, VD also exhibits multiple mechanisms of action in CP, including anti-inflammatory effects, immune regulation, the regulation of proliferation, and the induction of differentiation, apoptosis, and autophagy. More details can be found in Zheng M & Gao R’s excellent review [35].

#### 4.1.9. Isoliquiritigenin

Isoliquiritigenin (ILG), a simple chalcone-type flavonoid isolated from licorice roots, has been reported to exhibit antioxidative, anti-inflammatory, and hepatoprotective properties. ILG has been reported to suppress adipose tissue inflammation and attenuate high-fat diet-induced adipose tissue fibrosis by targeting innate immune sensors [99,100,101]. Recent studies have found that ILG significantly attenuates pancreatic fibrosis and inflammation by inhibiting the activation of PSCs and macrophage pancreatic infiltration [64]. Further studies showed that ILG inhibited the activation of human pancreatic stellate cells by negatively regulating ERK1/2 and JNK1/2 activity and related signaling pathways [64]. In summary, this evidence suggests that ILG may be a potential therapeutic agent to alleviate pancreatic fibrosis in CP.

#### 4.1.10. Puerarin

Puerarin is the most important active flavonoid component in the Chinese herb *Radix Puerariae*. Puerarin has a variety of pharmacological effects, including inhibiting platelet aggregation; antioxidant, anti-inflammatory, and diuretic properties; regulating blood pressure, blood sugar, and lipids; and protecting the myocardium; it is also virtually non-toxic. In recent years, puerarin has been well recognized for its anti-fibrotic effects in various fibrotic diseases such as liver, lung, kidney, and heart fibrosis [102,103,104,105]. A recent study demonstrated that puerarin has a significant inhibitory effect on the proliferation, migration, and activation of PSCs, as well as the phosphorylation of JNK1/2, ERK1/2, and p38 MAPK, suggesting that puerarin may be a potential candidate drug for treating CP, and the MAPK pathway may be its key target [66].

#### 4.1.11. Small Molecule Kinase Inhibitors and Synthetic Drugs

In addition to natural plant compounds, several small molecule kinase inhibitors and synthetic drugs also show anti-fibrotic activity [106,107,108]. Fibromodulin (FMOD) is increased in CP and is an important downstream mediator of oxidative stress. Inhibitors of ERK and JNK can reduce FMOD, thereby reducing PSCs activation [109]. Dasatinib, an inhibitor of several tyrosine kinases, has been proven to have a possible anti-fibrotic effect on CP and to reduce pancreatic fibrosis and macrophage infiltration [67]. As a PARPγ inhibitor, olapanib shows an anti-fibrotic effect in CP induced by cerulein [110]. As an inhibitor of discoidin domain receptor 1 (DDR1) and DDR2, imatinib can reduce ECM deposition and PSCs activation, as well as inhibit the TGF-β1/Smad pathway [68]. HDAC inhibitors are a new class of drugs that regulate chromatin structure and gene expression, and induce growth inhibition, apoptosis, and differentiation. Among these, Vorinostat (SAHA) and Trichostatin A (TSA) attenuate pancreatic fibrosis and PSCs apoptosis by increasing the expression of miR-15 and miR-16 to induce PSCs apoptosis and inhibit the inflammatory response [72]. Briefly, small molecule kinase inhibitors are one of the current research hotspots and show promising applications in the anti-fibrosis treatment of CP.

In vitro and in vivo studies showed that in human PSCs and nude mice with subcutaneous pancreatic cancer, metformin for type II diabetes decreased α-SMA, TGF-β1, and collagen expression in pancreatic tissue and inhibited the proliferation of PSCs by upregulating p-AMPK [69,70]. Furthermore, metformin also enhanced the sensitivity of PSCs and pancreatic cancer cells to gemcitabine treatment.

Yttrium oxide nanoparticles (NY) are unique antioxidants with significant anti-inflammatory activity. A recent study demonstrated that NY intervention significantly reduced the L-Arg-induced overexpression of α-SMA, N-Cadherin, MMP2, and TIMP1 in PSCs, indicating that NY inhibited the activation of PSCs [71]. Furthermore, NY exhibits the antifibrotic potential through the inhibition and modulation of PSCs activation, EMT, ECM aggregation, the TGF-β pathway, and inflammation [71]. 

Follistatin is an endogenous binding protein of activin A. The study revealed that activator A activates cs PSCs in an autocrine manner to promote collagen secretion and works in conjunction with TGF-β to promote the secretion and mRNA expression of PSCs [73]. In addition, follistatin can inhibit the secretion of TGF-β and collagen in PSCs and reduced α-SMA content [73].

The Chinese herb Chaihu Guizhi Ganjiang Decoction (CGGD) was found to alleviate pancreatic injury, reduce collagen deposition, and inhibit PSCs activation in CP rats [74]. Furthermore, CGGD promotes the phosphorylation of mTOR and JNK in pancreatic tissue and PSCs by downregulating Atg5, Beclin-1, and LC3B [74]. These results suggest that CGGD may inhibit PSCs activation and alleviate pancreatic fibrosis by inhibiting autophagy through the JNK/mTOR signaling pathway.

### 4.2. Antioxidant Therapy

As previously mentioned, oxidative stress is one of the most important mechanisms in the pathogenesis of CP, and targeting PSCs to counteract cellular oxidative stress is a therapeutic strategy worth considering. It was found that the blood levels of antioxidants in patients with alcoholic CP were insufficient, despite a controlled dietary intake [111]. In a randomized trial, compared to placebo, patients taking antioxidants showed a significant improvement in pain [112]. In a meta-analysis of eight studies, patients with CP who received antioxidant therapy experienced significantly less pain compared to the controls [113]. In another experiment, antioxidant NY significantly reduced lipid peroxidation and nitrite levels, upregulated tissue glutathione, and decreased iNOS and nitrotyrosine expression in L-Arg induced CP rats [71]. The natural plant compounds in the aforementioned anti-fibrosis therapy also exhibit antioxidant activity. For example, injecting emodin into rats with acute pancreatitis revealed a significant increase in pancreatic acinar cell apoptosis [114], which may be related to a decrease in DNA binding to NF-κB in pancreatic tissue and the subsequent inhibition of pro-inflammatory cytokines [115]. In addition, emodin inhibits the HTRA1/TGF-β/NF-κB signal cascade and promotes cell apoptosis through the calcium-mediated caspase-12 pathway [116,117,118,119]. In a rat pancreatitis model, curcumin reduced the severity of the disease, inhibited NF-κB activation, and decreased the mRNA levels of Il-6, TNF-α and iNOS in the pancreas [49]. In addition, the macrophage or monocyte infiltration in rats treated with ellagic acid and the ROS production in isolated PSCs were significantly reduced [59]. Resveratrol blocks the ROS-induced activation, invasion, migration, and glycolysis of PSCs by inhibiting the expression of miR-21 and increasing the protein levels of phosphatase and PTEN [52]. ILG is effective in reducing the severity of acute pancreatitis by inhibiting oxidative stress and regulating the Nrf2/HO-1 pathway [65]. The Nrf2-Keap1 signaling pathway is a key strategy to combat chronic inflammation [120]. In a rat pancreatitis model, an effective Nrf2 activator, dimethyl fumarate, demonstrated the upregulation of antioxidants in vitro and showed a significant ability to reduce inflammation and pancreatic damage in vivo compared to the controls [121]. However, it should be noted that few trials have been conducted with sufficient ability to demonstrate the significant efficacy of naturally occurring antioxidants in the treatment of pancreatitis [122]. Therefore, more experiments are needed to support the role of natural antioxidants in the treatment of CP.

### 4.3. Gene Therapy

The latest high-throughput sequencing technology, and even single-cell technology, allows for the accurate analysis of the abundance of mRNA expression and the expression profile in specific organs, tissues, and cells, enabling precise therapy at the gene level. Currently, miRNA and siRNA have been widely used in gene therapy. Several studies have shown that miRNA and siRNAs exhibit potential therapeutic value by targeting and inhibiting specific genes to inhibit the occurrence and progression of CP [123].

For example, this study showed that 5-FU-miR-15a inhibits the expression of YAP1 and BCL-2 in murine and human PSCs in the in vitro setting. Importantly, 5-FU-miR-15a suppresses PSCs proliferation and migration, and inhibits the invasion of pancreatic cancer cells [75]. Furthermore, the protein 3 (NLRP3) inflammasome containing the NACHT, LRR, and PYD domains is directly involved in PSCs activation in vivo and in vitro. The expression of PSCs activation markers α-SMA, collagen I, and fibronectin was reduced with NLPR3 siRNA [76]. In addition, activated YAP promotes PSCs proliferation, and siRNA reduces PSCs activation and fibrosis in CP animal models by knocking down YAP [77]. However, these studies are still in the laboratory stage and must be further validated in clinical trials.

## 5. Conclusions

Although the pathogenesis of CP has not been fully elucidated, activated PSCs have been widely recognized as key gatekeepers of pancreatic fibrosis, which is an important hallmark of CP, as well as in its associated disease, pancreatic cancer. Many prevention or treatment strategies targeting PSCs have been proposed for CP and fibrosis mediated by PSCs activation. Various antioxidant and gene therapies have emerged, and anti-fibrosis therapies in particular have been widely recognized, showing great promise for clinical application. In addition, immunotherapy targeting the PSCs may be expected to become a rising star in the treatment of CP. Despite the inclusion of the above potential pharmacological treatments for CP, there is a lack of relevant clinical trials regarding these treatments. It can be foreseen that with the in-depth disclosure of the pathogenesis of CP and the combination of different treatment strategies, the prevention and treatment of CP and its related diseases will be greatly improved.

## Figures and Tables

**Table 1:** Therapeutic strategies for chronic pancreatitis targeting pancreatic stellate cells.

Phytochemicals	Chemical Structures	Strategies	Main Effects
Curcumin	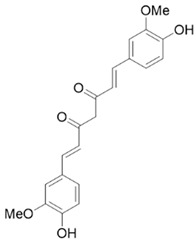	Anti-fibrosis	Inhibiting PDGF-induced proliferation of PSCs [47]; inactivating PSCs by inhibiting their proliferation [48]; inhibiting the levels of α-SMA, type I collagen, and fibronectin in TGF-β-activated primary PSCs [12].
		Antioxidant	Inhibiting NF-κB activation and decreasing the mRNA levels of Il-6, TNF-α, and iNOS in the pancreas [49].
Resveratrol	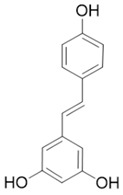	Anti-fibrosis	Inducing apoptosis and attenuating fibrosis by promoting caspase-3 activation [50]; inhibiting the marker of PSCs activation (α-SMA, collagen I, and fibronectin) by NF-κB signaling [51].
		Antioxidant	Blocking ROS-induced activation, invasion, and migration of PSCs by inhibiting miR-21 [52].
Rhein	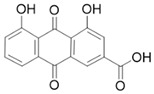	Anti-fibrosis	Attenuating PSCs activation, inhibiting α-SMA, fibronectin, and MMP in PSCs by modulating the SHH pathway [53,54].
Emodin	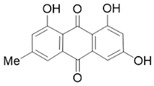	Anti-fibrosis	Reducing primary PSCs viability, downregulating the expression of fibrosis markers α-SMA, fibronectin, and collagen I [51].
EGCG	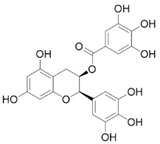	Anti-fibrosis	Inhibiting PDGF-induced PSCs proliferation and migration [55]; inhibiting the ethanol-induced activation of PSCs and the transformation of the myofibroblast-like phenotype [56,57].
Ellagic acid	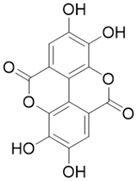	Anti-fibrosis	Inactivating PSCs by reducing α-SMA and ECM type I and III procollagen, preventing the transformation of PSCs from quiescence to a myofibroblast-like phenotype [58]; inhibiting PDGF-BB-induced PSCs proliferation and migration [59].
		Antioxidant	Inducing macrophage or monocyte infiltration, reducing ROS production in PSCs [59].
Eruberin A	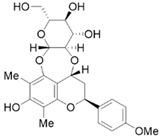	Anti-fibrosis	Suppressing gene expression of major fibrotic filaments and ECM mediators, inhibiting the activation of the PI3K/AKT pathway associated with the downstream cascade of inflammation and fibrogenesis [60].
VA and derivatives	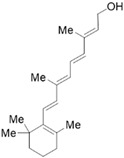 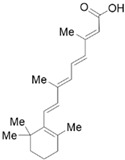	Anti-fibrosis	Inhibiting the proliferation and suppression of PSCs, producing ECM, and inducing apoptosis; its analogs can inhibit PSCs activation in pancreatic ductal adenocarcinoma [61].
VD and derivatives	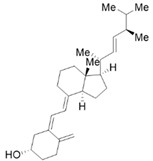 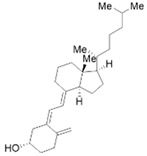 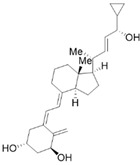	Anti-fibrosis	Inhibiting PSCs activation and reducing ECM deposition; its analogs may restart the quiescent state of the PSCs and reduce fibrosis [62,63].
ILG	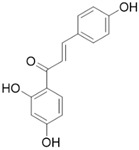	Anti-fibrosis	Inhibiting the activation of PSCs and macrophage pancreatic infiltration [64].
		Antioxidant	Inhibiting oxidative stress and regulating the Nrf2/HO-1 pathway [65].
Puerarin	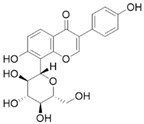	Anti-fibrosis	Inhibiting the proliferation, migration, and activation of PSCs [66].
Dasatinib	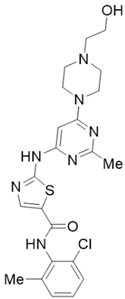	Anti-fibrosis	Reducing pancreatic fibrosis and macrophage infiltration [67].
Imatinib	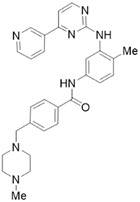	Anti-fibrosis	Reducing ECM deposition and PSCs activation; inhibiting the TGF-β1/Smad pathway [68].
Metformin	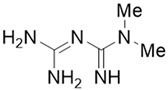	Anti-fibrosis	Inhibiting the proliferation of PSCs by upregulating p-AMPK [69,70].
NY	-	Anti-fibrosis Antioxidant	Inhibiting lipid peroxidation and decreased iNOS; inhibiting PSCs activation [71].
HDAC inhibitors	-	Anti-fibrosis	HDAC inhibitors promote the apoptosis of pancreatic stellate cells by upregulating miR-15/16 and disrupting TGF-β/Smad signaling for anti-fibrosis [72]
Follistatin	-	Anti-fibrosis	Follistatin can inhibit PSCs activation and collagen secretion by blocking autocrined activin A and decreasing TGF-β expression and the secretion of PSCs [73].
CGGD	-	Anti-fibrosis	Inhibiting PSCs activation, reducing collagen deposition [74].
5-FU-miR-15a	-	Gene therapy Anti-fibrosis	Inhibiting the proliferation and migration of PSCs; reducing RNA and protein expression of Yap1 and Bcl2 [75].
NLPR3 siRNA	-	Gene therapy Anti-fibrosis	Inhibiting the expression of PSCs activation markers α-SMA, collagen I, and fibronectin [76].
YAP siRNA	-	Gene therapy Anti-fibrosis	Reducing PSCs activation and fibrosis [77].

Note: EGCG, epigallocatechin gallate; VA, vitamin A; VD, vitamin D; ILG, isoliquiritigenin; NY, yttrium oxide nanoparticles; CGGD, Chaihu Guizhi Ganjiang Decoction.

## Data Availability

No new data were created or analyzed in this study. Data sharing is not applicable to this article.
